# Accuracy of pedigree and genomic predictions of carcass and novel meat quality traits in multi-breed sheep data assessed by cross-validation

**DOI:** 10.1186/1297-9686-44-33

**Published:** 2012-11-12

**Authors:** Hans D Daetwyler, Andrew A Swan, Julius HJ van der Werf, Ben J Hayes

**Affiliations:** 1 Biosciences Research Division, Department of Primary Industries, Bundoora, Victoria, 3083, Australia; 2 Cooperative Research Centre for Sheep Industry Innovation, Armidale, NSW, 2351, Australia; 3 Animal Genetics and Breeding Unit (AGBU), University of New England, Armidale, NSW, 2351, Australia; 4 School of Environmental and Rural Science, University of New England, Armidale, NSW, 2351, Australia; 5 La Trobe University, Bundoora, Victoria, 3086, Australia

## Abstract

**Background:**

Genomic predictions can be applied early in life without impacting selection candidates. This is especially useful for meat quality traits in sheep. Carcass and novel meat quality traits were predicted in a multi-breed sheep population that included Merino, Border Leicester, Polled Dorset and White Suffolk sheep and their crosses.

**Methods:**

Prediction of breeding values by best linear unbiased prediction (BLUP) based on pedigree information was compared to prediction based on genomic BLUP (GBLUP) and a Bayesian prediction method (BayesR). Cross-validation of predictions across sire families was used to evaluate the accuracy of predictions based on the correlation of predicted and observed values and the regression of observed on predicted values was used to evaluate bias of methods. Accuracies and regression coefficients were calculated using either phenotypes or adjusted phenotypes as observed variables.

**Results and conclusions:**

Genomic methods increased the accuracy of predicted breeding values to on average 0.2 across traits (range 0.07 to 0.31), compared to an average accuracy of 0.09 for pedigree-based BLUP. However, for some traits with smaller reference population size, there was no increase in accuracy or it was small. No clear differences in accuracy were observed between GBLUP and BayesR. The regression of phenotypes on breeding values was close to 1 for all methods, indicating little bias, except for GBLUP and adjusted phenotypes (regression = 0.78). Accuracies calculated with adjusted (for fixed effects) phenotypes were less variable than accuracies based on unadjusted phenotypes, indicating that fixed effects influence the latter. Increasing the reference population size increased accuracy, indicating that adding more records will be beneficial. For the Merino, Polled Dorset and White Suffolk breeds, accuracies were greater than for the Border Leicester breed due to the smaller sample size and limited across-breed prediction. BayesR detected only a few large marker effects but one region on chromosome 6 was associated with large effects for several traits. Cross-validation produced very similar variability of accuracy and regression coefficients for BLUP, GBLUP and BayesR, showing that this variability is not a property of genomic methods alone. Our results show that genomic selection for novel difficult-to-measure traits is a feasible strategy to achieve increased genetic gain.

## Background

Sheep meat production is increasing and replacing wool production as the primary product of the Australian sheep industry [1]. Improving growth traits through selection for increased live and a carcass weight is an important driver of profitability. Providing consistently high-quality meat is also essential to maintain high consumer acceptance and depends on several carcass quality criteria, such as intra-muscular fat, shear force and Omega-3 content [1,2]. Genomic selection [3] is applied in an ever growing number of livestock species, e.g. [4-8], and could increase economic returns from lamb production [9]. Genomic selection can be applied at a number of well-known entry points of breeding schemes to increase genetic progress. Genomic estimated breeding values (GEBV) can be obtained for selection candidates at a young age before phenotypic information is available and be used to increase accuracy of selection and shorten generation intervals. This is useful for traits measured later in life, such as adult greasy fleece weight and reproduction and in cases when phenotypic evaluation involves invasive or destructive approaches such as for carcass composition and meat quality, which are traditionally measured on the relatives of selection candidates.

Estimating GEBV for selection candidates requires a reference population with both marker genotypes and phenotypes. Because selection candidates often lack phenotypic records, the predictive performance of GEBV can be assessed either with a set of validation individuals that have highly accurate EBV, e.g. sires with many progeny [4,10], or by cross-validation, e.g. [6,11-13]. Both validation methods require that the validation population and the potential selection candidates have a similar genetic make-up, such that the accuracies obtained for the selection candidates will reflect those calculated using validation individuals. In particular, the validation and selection individuals should have similar relationships to the reference population [14-16].

For difficult-to-measure and novel traits, individuals with highly accurate EBV often do not exist. Thus, in such cases, cross-validation is applied. In the cross-validation approach, the reference population is divided into a number of subsets and each subset is predicted using a reference population that excludes this particular subset. The method used to divide the data has been shown to affect prediction accuracy, e.g. [6,17,18]. Choosing fully random subsets is the simplest implementation but this ignores data structures, for example presence of sire half-sib groups. Several studies have divided subsets randomly with constraints on data structure, such as age [17], family [18], and relatedness [6]. Another consideration is the size of the subsets used for cross-validation. The larger the subset, the smaller the sampling variance of the correlation between predicted and observed variables is expected to be [11,19]. However, larger subsets decrease the size of the reference population, resulting in a trade-off between the size of subsets and the accuracy achieved.

The utility of applying genomic prediction must be evaluated against what would be achieved with non-genomic approaches, such as best linear unbiased prediction (BLUP) using pedigree [20]. Cross-validation studies have compared accuracies of EBV using traditional pedigree methods and accuracies of GEBV [6,18] but these comparisons have not been made for multi-breed livestock data. Another point to consider is the phenotype used to estimate accuracies in cross-validation studies. Most studies correlate with phenotypes but the accuracies resulting from these comparisons may be affected by fixed effects that are often included in the prediction models.

The aim of this study was to predict GEBV for several carcass and novel meat quality traits in a multi-breed sheep population. A previous study in the same population investigated how much of the accuracy of GEBV could be attributed to population structure [21]. Here, an across sire family cross-validation scheme was used to estimate accuracies of GEBV in several sheep breeds and their crosses. GEBV were obtained with three methods: BLUP, genomic BLUP (GBLUP) and BayesR. In addition, accuracies were calculated based on phenotypes or adjusted phenotypes and with or without adding a polygenic effect.

## Methods

**Figure 1:**
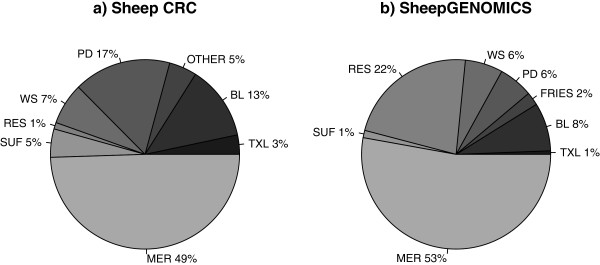
**Proportions of different breeds in the two datasets.** Texel:TXL, Border Leicester: BL, Polled Dorset: PD, White Suffolk: WS, research lines: RES, Suffolk: SUF, East Friesian: FRIES and Merino: MER.

Datasets from the Cooperative Research Centre for Sheep Industry Innovation (CRC, genetically connected flocks in 8 locations) [22] and SheepGENOMICS (SG, one flock) [23] were combined to increase the size of the reference population. Different strategies were used to sample the rams and determine the number of progeny per ram in CRC and SG. Rams used for breeding in the CRC flocks were sampled from the general Australian sheep population to maximise connectedness and sire progeny groups contained approximately 40 animals. In contrast, the SG project was initially set up as a linkage study with 20 sires and large progeny groups. Depending on the traits examined, the combination of these datasets resulted in various sizes of reference populations (animals with both phenotypes and genotypes) for genomic analyses, ranging from 3107 to 8075 animals (Table 1). The breed content of the reference population for hot carcass weight is shown in Figure 1 and is representative of other traits because hot carcass weight was used as a covariate in most analyses. Both reference populations had a significant proportion of Merino individuals and only this breed had a substantial proportion of purebred animals. Most other individuals were crossbreds of meat breed sires and Merino or Merino/Border Leicester dams.

**Table 1:** **Summary statistics and heritabilities (***h*^*2*^**) for the phenotypic traits analysed for the two data sets (CRC and SG)**

	**CRC**			**SG**			**Total**	
**Trait, units**	**Mean**	**SD**	**N**	**Mean**	**SD**	**N**	**N**	***h***^***2***^
EMD, mm	29.9	3.8	5117	24.7	3.4	2119	7236	0.25
FAT, mm	4.0	2.3	4973	2.7	8.5	2100	7073	0.40
HCWT, kg	22.7	3.6	5170	18.3	2.7	2127	7297	0.59
DRESS, %	45.4	3.6	4974	43.7	3.0	2111	7085	0.34
LMY, %	58.0	3.1	5964	36.6	1.9	2111	8075	0.32
IMF, %	4.2	1.0	4644	3.4	1.5	683	5327	0.49
IRON, mg/kg	20.1	3.7	4618	-	-	-	4618	0.29
DPA, mg/100g	48.3	15.9	3109	-	-	-	3109	0.24
EPA, mg/100g	23.6	9.3	3107	-	-	-	3107	0.26

Heritability (*h*^2^) estimated in this dataset, CRC is Sheep CRC dataset, SG is SheepGenomics dataset.

The following traits were analysed and phenotypic information for these traits is provided in Table 1. Carcass eye muscle depth (EMD, mm), carcass fat depth at site C (FAT, mm, depth of fat at maximum EMD), hot carcass weight (HCWT, kg), dressing percentage (DRESS, %), calculated as the ratio of HCWT to pre-slaughter weight, intra-muscular fat (IMF, %), iron content of wet muscle tissue (IRON, mg/kg ), and the concentration of omega 3 fatty acid compounds eicosapentaenoic acid (EPA, mg/100g) and docosapentaenoic acid (DPA, mg/100g). Lean meat yield (LMY, %) was estimated on the CRC animals by a combination of other carcass traits and validated by computed tomography (CT) scanning [24]. On the SG animals, LMY was computed as the ratio of HCWT and actual lean meat after bone-out [23]. To account for these differences in methodology, LMY was standardised (mean = 0, standard deviation (SD) = 1) within the CRC and SG datasets before the datasets were merged.

All animals were genotyped using the Illumina 50K ovine SNP chip, containing 54 977 single nucleotide polymorphisms (SNP) (Illumina Inc., San Diego, USA). After applying the following quality control measures, 48 599 SNP were retained: SNP were removed if the call rate was less than 95%, if the Illumina Gentrain score was less than 0.6, if the minor allele frequency was less than 0.01, if the SNP was not in Hardy-Weinberg equilibrium (a *P*-value cut-off of 1×10^–15^), if the genome location was unknown or if the SNP showed complete linkage disequilibrium (r^2^ > 0.99) with another SNP on the chip. Data for a genotyped animal were removed if the genotype call rate was less than 0.9 for that animal or if the animal’s mean heterozygosity was higher than 0.5, indicating sample contamination. The genotype database was built over a number of years, missing genotypes were initially imputed using fastPHASE [25] and more recently, missing genotypes were imputed using Beagle [26], after this program became available.

Three analysis methods were used. Non-genomic breeding values were predicted after estimating variance components using pedigree information and restricted maximum likelihood (REML), using the following single-trait model [27]:

(1) $$y=1\mu +Xb+Z_{1}a+Z_{1}Qq+e$$

where **y** is a vector of phenotypic records, **X**, and **Z**_**1**_ are design matrices relating the fixed and random effects to the phenotype, **Q** is a matrix containing breed proportions for each animal, derived from pedigree information, **μ** is the mean, **b** is a vector of fixed effects, **a** is a vector of random additive polygenic effects, **q** is a vector of random breed effects, fitted as partial regressions, and **e** is the vector of residuals. The following distributions were assumed: **a** ~ *N* (0,*σ*_*a*_^2^**A**), **q** ~ *N* (0,*σ*_*q*_^2^**I**), and **e** ~ *N* (0,*σ*_*e*_^2^**I**), where **A** is the numerator relationship matrix, *σ*_*a*_^2^ is the additive variance, *σ*_*q*_^2^ is the variance of breed effects, and *σ*_*e*_^2^ is the residual variance. The base model included the following fixed effects: sex, birth type, rearing type, contemporary group (birth year × site × slaughter group), and age at trait recording. Age of the dam was fitted only for CRC data. HCWT was included as a fixed covariate for all traits except for DRESS and LMY. The size of the relevant pedigree was 16 985 individuals. The phenotypes (y) were restricted to genotyped animals to make a fair comparison to the genomic prediction methods.

Genomic breeding values were calculated using GBLUP, for which variance components were also estimated with REML analysis [27], using the model:

(2) $$y=1\mu +Xb+Z_{1}a+Z_{2}g+Z_{1}Qq+e$$

where **Z**_**2**_ is a design matrix, **g** is a vector of random additive genomic effects distributed as *N* (0,*σ*_*g*_^2^**G**), *σ*_*g*_^2^ is the genomic variance, **G** is the genomic relationship matrix [28]. SNP with allele frequencies less than 0.005 were removed from the calculation of **G** to improve numerical stability. Phenotypes, rather than de-regressed estimated breeding values, were used to ensure independence of reference and validation sets. If all phenotypes were used to calculate breeding values, then the accuracy of predicting an animal without a phenotype would be overestimated, because phenotypes of validation animals contributed to the reference pedigree breeding values.

A Bayesian genomic prediction method using a mixture of four normal distributions with increasing variance for marker effects (BayesR) [29] was implemented in two steps. First, phenotypes were fitted using model 1 but without a polygenic effect (**y** = **1μ** + **Xb** + **Z**_**1**_**Qq** + **e**). The resulting adjusted phenotypes (y*) or residuals were then analysed using BayesR in model:

(3) $$y^{*}=1\mu +Z_{1}a+Wm+e$$

where **W** is a design matrix relating adjusted phenotypes to random marker effects (**m**). BayesR is described in more detail in [29]. Briefly, marker variances can come from distributions with variances *σ*_1_^2^= 0, *σ*_2_^2^= 0.0001*σ*_*g*_^2^, *σ*_3_^2^= 0.001*σ*_*g*_^2^, or *σ*_4_^2^= 0.01*σ*_*g*_^2^, and starting values for *σ*_*g*_^2^ were from GBLUP analysis. The prior for the proportion of markers in each distribution was drawn from a Dirichlet distribution. Priors for other parameters were chosen as in Erbe et al. [29]. Ten parallel chains of 50 000 iterations (20 000 burn-in) were run for each subset.

Posterior means of marker effects of BayesR resulting from post burn-in chains were averaged across chains and replicates and then standardised by dividing them by the standard deviation of the adjusted phenotypes (SD). SNP with effects greater than 0.005 SD were (arbitrarily) chosen and potential candidate genes were searched for on http://www.livestockgenomics.csiro.au/cgi-bin/gbrowse/oarv2.0/ using a 1 Mb interval with 0.5 Mb on each side of the SNP. The probability of the effect of a SNP being in the largest distribution (*σ*_4_^2^) was also investigated.

Performance of genomic prediction was evaluated using cross-validation. It is unlikely that potential selection candidates in the Australian sheep population have full or half-sibs in the reference population. Thus, entire sire families in the CRC dataset were randomly chosen and combined into subsets of approximately 500 individuals (CRC subsets). Thus, genomic predictions were evaluated across sire families and larger reference populations had more subsets. The SG dataset was not divided into subsets but was added to each reference population. The performance of predictions was not evaluated for the SG data, because this population is not expected to be representative of the general sheep population. Genomic predictions were calculated for each CRC subset, with the reference set consisting of all other CRC subsets and the SG dataset. Accuracy was evaluated in each validation subset as the Pearson correlation of genomic predicted breeding values ( $\hat{g}$) or genomic plus polygenic predicted breeding values ( $\hat{g}$ + $\hat{a}$) with either phenotypes (**y**) or adjusted phenotypes (y*). Accuracies were divided by $\sqrt{h^{2}}$ from model 1 to adjust for the upper limit of accuracy of a phenotype/residual. The bias of breeding values (both $\hat{g}$ and $\hat{g}$ + $\hat{a}$) was calculated as the regression of phenotypes or adjusted phenotypes on predicted breeding values. Accuracy and bias were calculated for the whole validation subset and for each subdivision of each subset by the sire breeds Merino (MER, effective population size, *N*_*e*_ ~ 850), Border Leicester (BL, *N*_*e*_ ~ 250), Polled Dorset (PD, *N*_*e*_ ~ 300), and White Suffolk (WS, *N*_*e*_ ~ 300) [30].

Genetic relatedness of validation animals with the reference population was calculated for each subset as the mean of the squared relationships between validation and reference animals and mean of the top 10 relationships. Other studies have concluded that these measures are more predictive of accuracy than mean relationships, hence the latter was not reported [15,16].

## Results and discussion

**Figure 2:**
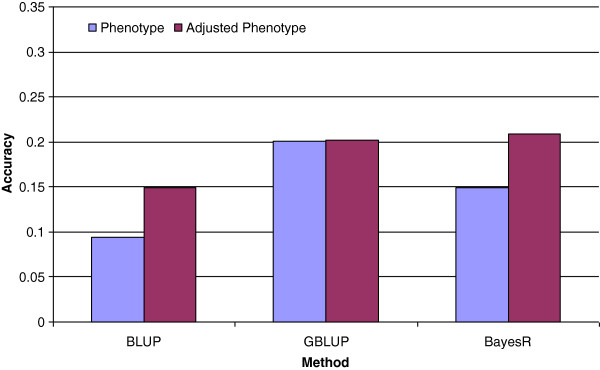
**Mean accuracy across all traits and breed groups of BLUP, GBLUP and BayesR.** Accuracy was calculated as the correlation between predicted breeding values and phenotypes or adjusted phenotypes.

Use of genomic data increased the accuracies of prediction, depending on the trait (mean accuracy across traits = 0.2) and both GBLUP and BayesR led to more accurate GEBV than pedigree-based BLUP (Figure 2). Increases in accuracy were greater when based on correlations with phenotypes adjusted for fixed and breed effects than when based on correlations with unadjusted phenotypes for pedigree-based BLUP and BayesR but not for GBLUP. Correlations with adjusted phenotypes varied less between breeds [see Additional file 1: Table S1], e.g. for GBLUP for the trait DRESS. Similarly, when correlations were calculated using the whole subset (i.e. without subdividing by breed), correlations of GEBV with phenotypes were often much higher than with adjusted phenotypes, although this trend was more pronounced with GBLUP than with BayesR. This confirms that estimating accuracies with unadjusted phenotypes involves a component related to fixed effects or breed, even when these are fitted when computing GEBV. Both genomic methods used in this study included a polygenic effect. Adding the estimate of the polygenic effect to the GEBV resulted in a small increase in accuracy (about 1%, data not shown). Furthermore, GBLUP was also run without a polygenic effect and no significant difference in accuracy was observed compared to GBLUP with a polygenic effect but without adding the estimate of the polygenic effect when computing the GEBV. In the next section, only accuracies based on correlations of GEBV plus estimates of polygenic effects with adjusted phenotypes are reported because they were more balanced between breeds and seemed less confounded with fixed effects.

**Figure 3:**
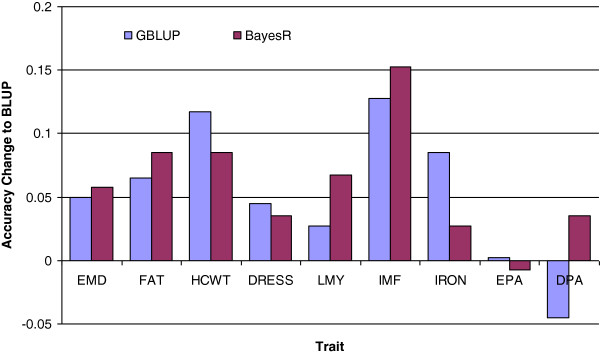
Change in mean accuracy of across breeds and traits of GBLUP and BayesR when compared to BLUP.

The increase in accuracy with genomic methods over BLUP was not uniform across traits (Figure 3). While genomic methods increased the accuracy of IMF substantially, accuracies were similar and closest to those from pedigree-based BLUP for the traits EPA and DPA.

**Figure 4:**
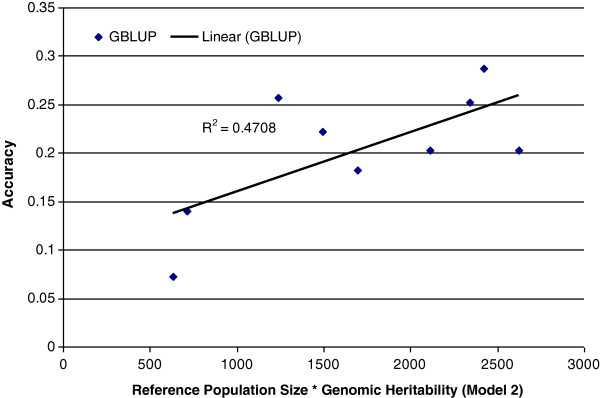
Relationship between mean accuracy across breeds and product of reference population size by genomic heritability (Model 2).

Figure 4 illustrates the relationship between genomic prediction accuracy and the product of reference set size and genomic heritability (*R*^*2*^ = 0.47). The results show a clear trend of increased accuracy with increasing size of the reference population and suggest that further increases in accuracy should be possible if the reference population is increased further.

**Figure 5:**
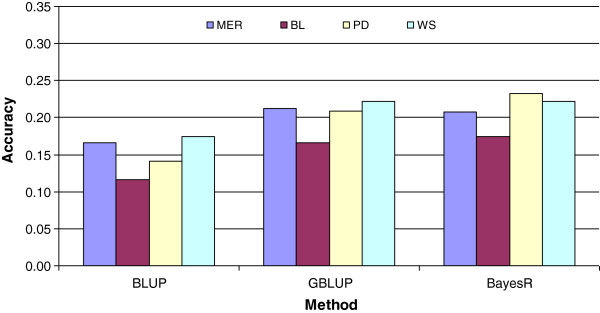
Mean accuracy across all 9 traits of BLUP, GBLUP and BayesR in Merino, Border Leicester, Polled Dorset, and White Suffolk sired sheep.

The accuracy of GEBV was lower in Border Leicester sire breed groups than in the Merino, Polled Dorset and White Suffolk breeds (Figure 5). This is probably because Border Leicester sheep have the lowest mean breed proportion in the reference set of the four validation breeds. For the Merino, Polled Dorset and White Suffolk breeds, similar genomic accuracies were obtained, although the Merino breed represented the largest proportion in the reference population. The fact that the accuracy of GEBV for the Merino breed was not larger could be because the Merino population has a higher *N*_*e*_ (about 800) [30] than the Polled Dorset or White Suffolk populations (*N*_*e*_ ~ 300), which results in a larger number of independent chromosome segments to be predicted [31-33]. Thus, in the terminal breeds, the accuracy of GEBV was similar to that in the Merino breed, despite a smaller number of phenotypic records in the reference set, because of lower genetic diversity in those breeds. Another study [4], reported a higher genomic prediction accuracy for wool and weight traits in Merino than in terminal breeds using the same populations, but that is likely because the proportion of the reference population that is Merino is larger for those traits than for meat quality traits.

**Figure 6:**
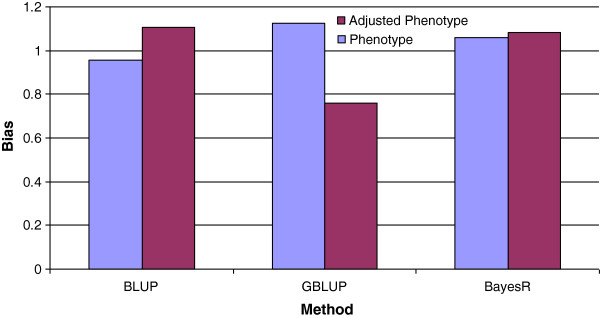
Mean bias across all traits and breed groups for BLUP, GBLUP and BayesR from regression of phenotypes or adjusted phenotypes on predicted breeding values.

The slope of regression of either phenotypes or adjusted phenotypes on GEBV was close to 1 for all methods, except when using adjusted phenotypes and GBLUP, which resulted in a regression coefficient of 0.78 (Figure 6 and Table S2 [see Additional file 1: Table S2]). Thus, overall, there is little evidence for bias in the predictions. Intercepts were close to 0 for all methods.

Table S3 [see Additional file 1: Table S3] contains the mean genetic relationships of validation with reference animals, calculated as the mean of the top 10 genomic relationships for each individual. Small differences in relatedness between breed groups and between traits were observed, ranging from 0.102 for DRESS to 0.168 for IRON, both in the Merino breed. No clear relationships of the mean genetic relationship with the achieved accuracy were found, for several reasons. First, the sampling variances of the correlations between GEBV and phenotypes were too large due to the small size of the validation sets [see Additional file 1: Table S4]. Secondly, the genomic relationship matrix used here was based on the original Yang et al. [28] implementation, which does not adjust for breed (base) allele frequencies. While these scaling issues are not likely to decrease predictive performance substantially, they will numerically affect mean relationships within a breed and may limit the possibility of finding a relationship between magnitude of mean relationships and within-breed accuracies. One possible solution would be to scale allele frequencies within breeds to their respective breed base allele frequencies before calculating the relationship matrix [29].

In the cross-validation design applied here, sires were chosen randomly and all their progeny were assigned to subsets. This prevented the upward bias of accuracies that would result from within-family prediction when half sib families are randomly split between reference and validation datasets. The accuracies obtained with our approach are expected to better reflect what would be achieved across a range of industry selection candidates with varying degrees of relationships to the reference animals. A further complication in our study was that the reference and validation populations were mostly made up of crossbreds, yet potential selection candidates in the industry are likely purebred individuals. Dividing the validation sets by sire breed groups was used to approximate the accuracy of purebred selection candidates. Because all animals have a large Merino component, the accuracies obtained with the Border Leicester, Polled Dorset and White Suffolk validation sets (sire breed groups) are not strictly equivalent to the accuracy which would be obtained with purebred animals. However, in the absence of purebred individuals with carcass data, this represents the best possible approximation. However, while the selection of breeding stock takes place among purebred individuals, commercial stock results from crosses between terminal and maternal sheep breeds.

Another aim of this study was to compare the variability of cross-validated EBV accuracies across subsets from pedigree-based BLUP and genomic methods. No large differences were observed, aside from the increase in accuracy using genomic data. In addition, accuracies from pedigree-based BLUP and genomic methods had very similar standard errors, indicating that cross-validation accuracies are just as variable across subsets for pedigree-based BLUP.

**Figure 7:**
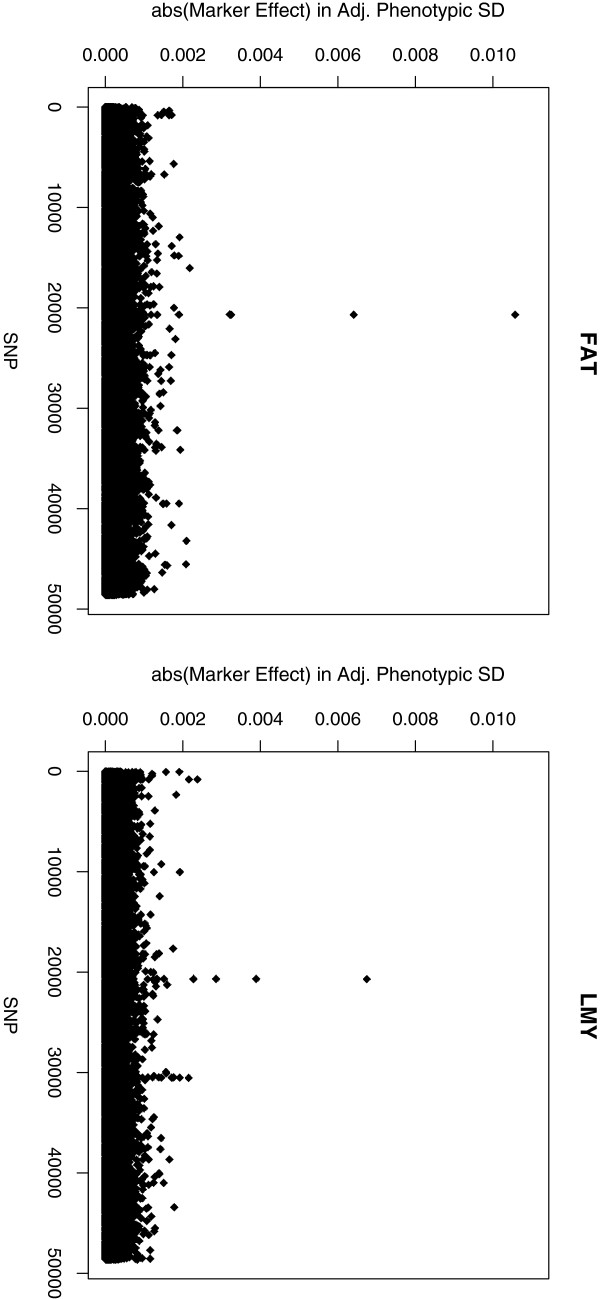
**Marker effects in standard deviations in carcass fat depth and lean meat yield.** The peaks for both traits are close to position 37 Mb on chromosome 6.

While BayesR did not increase the accuracy of genomic prediction compared to GBLUP, it does explicitly estimate marker effects and the proportion of markers in each of the four distributions. BayesR estimates of marker effects greater than 0.05 adjusted phenotypic trait SD are presented in Table 2. The largest estimated effect was observed for FAT (0.0106 SD, Figure 7) but most markers had very small effects. Thus, few large effects were detected in our analysis (Figure 7 and [see Additional file 1: Figure S1]). The magnitude of the estimated effects suggests that BayesR can still shrink large effects heavily, although it models four marker distributions, and this could partially explain the small number of large effects estimated or there just may be no true large marker effects in these traits. The SNP with the largest effects also tended to also have the highest probability of being in the largest SNP distribution (*σ*_4_^2^). In addition, the largest effects were consistently assigned to the same SNP across the 10 parallel BayesR chains.

**Table 2:** Information of SNP with greater than 0.5 SD effects, including all genes present with 0.5 Mb on either side

**Ch**	**Pos (bp)**	**SNP Name**	**Trait**	**Effect (SD)**	**Candidate Genes**
1	142,833,353	OAR1_154240036	HCWT	0.0081	ROBO2
1	274,065,940	OAR1_296010698	DRESS	0.0058	SATB1
3	58,809,386	OAR3_62808815	HCWT	0.0095	TSC21, EIF2AK3, RPIA, IGK, PSD4, IL1RN, IL1F10, IL1F5
3	60,199,488	OAR3_64213489	DRESS	0.0052	SLC20A1, CHCHD5, POLR1B, TTL, NCK2, Augurin, cDNA FLJ78230
6	36,877,942	OAR6_41003295	FAT	0.0064	PPM1K, ABCG2, PKD2, SPP1, MEPE, IBSP, LAP3, FAM182A, DCAF16, NCAPG, LCORL
6	37,228,504	s17946	FAT	0.0106	
6	37,228,504	s17946	IMF	0.0077	
6	37,228,504	s17946	DRESS	0.0060	
6	37,757,850	OAR6_41936490	LMY	0.0067	
11	20,696,610	OAR11_21345650_X	IMF	0.0058	CRYABA1, NYFIP2, TAOK1, GIT1, ANDKRD13B, SSH2, EFCAB5, NSRP1, SLC6A4, BLMH, CPD
14	2,380,675	OAR14_2924168	IMF	0.0070	Chymotrypsinogen A, BCAR1, CFDP1, CFDP2, TMEM170A, BVDV, ADAT1, KARS, CNTNAP4
14	17,972,633	s20405	IMF	0.0051	HEATR3, PAPD5, ADCY7, BRD7, NKD1, NX20, NOD2, CYLD

Full names of genes with locations are listed in Additional file 1, Table S5.

Potential candidate genes within a 1 Mb interval with 0.5 Mb on each side of the SNP are also presented in Table 2. One region on chromosome 6 contained SNP with estimated effects ranging from 0.0060 to 0.0106 SD for FAT, IMF, DRESS and LMY. Genes in this region included ATP-binding cassette sub-family G member 2 (*ABCG2)* and Polycystin-2 (*PKD2)*, which have been reported as having been under selection in an analysis of a large number of sheep breeds [30]. *ABCG2*, a gene involved in ATP binding, has been found to contain a causative mutation that affects milk yield and composition in dairy cattle [34] and has also been investigated as a candidate gene for facial eczema in sheep [35]. Another potential gene of interest is *NCK2* protein. This gene is close to SNP OAR3_64213489 (DRESS 0.0052SD) and codes for an adaptor protein that associates with tyrosine-phosphorylated growth-factor receptors. ARF GTPase-activating protein *(GIT1)*, which is close to SNP OAR11_21345650_X (IMF, 0.0058SD), codes for a GTPase-activating protein that is possibly involved in vesicle trafficking, adhesions and cytoskeletal organisation.

The main benefit of genomic selection for carcass and novel meat quality traits is that it is not necessary to sacrifice valuable selection candidates for testing, and GEBV can be obtained early in life. Genomic predictions can be trained within a set of industry representative individuals and then applied in the general sheep population. In its current form, this process is implemented in a centralised approach in sheep, in which test animals are housed in information nucleus flocks. However, data for training could also be collected during slaughter of industry stock, which could substantially increase the number of records. One advantage of an information nucleus is that animals are well identified and similarly managed, and fixed effects are fully recorded. Including industry records would require further investment in recording and tracking of animal production and movement and the development of uniform standards for measurement, sampling and testing at slaughter facilities.

One application of genomic selection is the prediction of accurate breeding values of juveniles without phenotypic records. This allows for significant shortening of generation intervals. In addition, some sheep breeders use juvenile in-vitro fertilised embryo transfer (JIVET), which consists of harvesting immature oocytes from 6 to 8 week old ewe lambs and implanting these into sexually mature individuals after *in vitro* fertilisation [36]. The combination of genomic selection and JIVET could be a powerful tool to increase genetic gain for novel meat traits. For example, lines with high omega-3 content or superior eating quality could be developed. The increase in genetic gain resulting from genomic selection would need to be combined with a strategy to limit a reduction in genetic diversity, such as using optimised contributions and mating schemes [37-40].

The accuracies of EBV obtained with genomic methods were not substantially higher than accuracies of EBV obtained with pedigree-based BLUP. Given the large reference population size, one could have expected a larger increase. However, in our case, many breeds contributed to the reference population and the limited increase in accuracy could be explained by small contributions obtained from across-breed prediction, which has been found to be very low in this population [21]. Increasing marker density, either through a high-density SNP chip or through next-generation sequencing, could increase accuracies from across-breed prediction. Simulation studies with simple genetic architectures have shown that using sequence data can be beneficial [41]. In contrast, in a perhaps under-powered study using empirical Drosophila sequence data, no increase in genomic prediction accuracy was observed compared to the use of lower SNP densities [42]. In dairy cattle, there is some evidence that across-breed prediction can be increased when using a high-density array [29]. A higher density SNP chip could potentially be more beneficial in sheep than in cattle, because the *N*_*e*_ is greater for most sheep breeds than in dairy cattle [30]. In Holstein cattle, 80% of the genetic variance was captured by the 50k bovine SNP chip [43]. Using the same methodology between 30 to 55% of the genetic variance was captured by the 50k ovine SNP chip in Merino sheep, depending on trait (results not shown). Thus, increasing marker density may result in substantial increases in accuracy, both within and across sheep breeds. Currently, the implementation of high-density arrays, and potentially sequence data, is accomplished using a two-step approach. First, reference populations that are genotyped at medium density (e.g. 50 000 SNP) are imputed up to higher density, using a sample of individuals that is genotyped at the higher density. Second, the imputed reference population is used for genomic prediction.

## Conclusions

Genomic predictions for meat quality traits in sheep are potentially valuable because they can be applied early in life and do not require potential selection candidates to be sacrificed. In a large multi-breed sheep dataset, genomic prediction resulted in greater accuracies of EBV than pedigree-based BLUP, but for some traits the increase in accuracy was small. Accuracy increased as reference population size increased and the accuracy was greater for the Merino, Polled Dorset and White Suffolk breeds than for the Border Leicester breed. The latter result is explained in part by the lower proportion of Border Leicester sheep in the reference population. It also suggests that across-breed prediction is limited with the 50k SNP chip. The methods GBLUP and BayesR produced very similar accuracies of GEBV, with a mean accuracy of approximately 0.2 across traits. Few markers with large effects were discovered but one region on chromosome 6 was associated with large effects for several traits. Validation correlations of GEBV with phenotypes adjusted for estimates of fixed effects were less variable than correlations with unadjusted phenotypes, and there was little evidence of bias in the GEBV. The general behaviour of cross-validation accuracies was very similar for pedigree-based BLUP, GBLUP and BayesR. In conclusion, genomic breeding values can provide a powerful tool to increase genetic progress in sheep, especially when combined with reproductive technologies.

## Competing interests

The authors declare that they have no competing interests.

## Authors’ contributions

HDD, JHJvdV and BJH designed the study, HDD and AAS analysed the data and HDD wrote the manuscript. All authors have read and approved the manuscript.

## Supplementary Material

Additional file 1 **Table S1.** Accuracies per trait and sire breed group of BLUP, GBLUP and BayesR, Accuracy calculated as r(EBV, observed variable)/h, where the observed variable was either a phenotype or adjusted phenotype. **Table S2.** Evaluation of bias per trait and sire breed group of BLUP, GBLUP and BayesR. Regression of observed variables on EBV, where the observed variable was either a phenotype or an adjusted phenotype. **Table S3.** Mean of top 10 genomic relationships of validation animals to reference population. **Table S4.** Number of validation animals (SE) per sire breed across subsets used for calculating accuracy, bias and relationships to reference. **Table S5.** Description of potential candidate genes within 0.5Mb of SNP with greater than 0.05 adjusted phenotypic SD effects. **Figure S1.** Plots of absolute marker effects in adjusted phenotypic SD for all traits. Click here for file
