# Supplementary material for: Accuracy of pedigree and genomic predictions of carcass and novel meat quality traits in multi-breed sheep data assessed by cross-validation
Source: Genet Sel Evol. 2012 Nov 12;44(1):33. doi: 10.1186/1297-9686-44-33 (PMC3506471; doi:10.1186/1297-9686-44-33)
Supplement: Additional file 1 — Table S1. Accuracies per trait and sire breed group of BLUP, GBLUP and BayesR, Accuracy calculated as r(EBV, observed variable)/h, where the observed variable was either a phenotype or adjusted phenotype. Table S2. Evaluation of bias per trait and sire breed group of BLUP, GBLUP and BayesR. Regression of observed variables on EBV, where the observed variable was either a phenotype or an adjusted phenotype. Table S3. Mean of top 10 genomic relationships of validation animals to reference population. Table S4. Number of validation animals (SE) per sire breed across subsets used for calculating accuracy, bias and relationships to reference. Table S5. Description of potential candidate genes within 0.5Mb of SNP with greater than 0.05 adjusted phenotypic SD effects. Figure S1. Plots of absolute marker effects in adjusted phenotypic SD for all traits. [file 1297-9686-44-33-S1.doc]

**Additional file 1:**

**Accuracy of pedigree and genomic predictions of carcass and novel meat quality traits in multi-breed sheep data assessed by cross-validation**

Hans D. Daetwyler, Andrew A. Swan, Julius H. J. van der Werf, and Ben J. Hayes

Table S1. Accuracies of BLUP, GBLUP and BayesR, calculated as r(GEBV + polygenic, observed variable)/h, where the observed variable was either a phenotype or adjusted phenotype.

| Trait | Method | Obs.Var. | ALL | MER | BL | PD | WS |
| --- | --- | --- | --- | --- | --- | --- | --- |
| EMD | BLUP | Phenotypes | 0.27(0.05) | 0.15(0.08) | 0.06(0.15) | 0.07(0.08) | 0.24(0.08) |
|  |  | Adj. phenotype | 0.17(0.04) | 0.24(0.05) | 0.09(0.09) | 0.15(0.06) | 0.21(0.06) |
|  | GBLUP | Phenotypes | 0.52(0.06) | 0.02(0.07) | 0.15(0.12) | 0.19(0.10) | 0.18(0.11) |
|  |  | Adj. Phenotype | 0.20(0.04) | 0.22(0.08) | 0.21(0.09) | 0.26(0.08) | 0.20(0.09) |
|  | BayesR | Phenotypes | 0.27(0.05) | 0.02(0.07) | 0.07(0.11) | 0.26(0.09) | 0.12(0.10) |
|  |  | Adj. Phenotype | 0.23(0.04) | 0.23(0.09) | 0.18(0.09) | 0.29(0.08) | 0.22(0.09) |
|  |  |  |  |  |  |  |  |
| FAT | BLUP | Phenotypes | 0.08(0.03) | 0.06(0.04) | 0.12(0.10) | 0.12(0.05) | 0.02(0.08) |
|  |  | Adj. Phenotype | 0.12(0.03) | 0.14(0.04) | 0.16(0.08) | 0.05(0.06) | 0.12(0.06) |
|  | GBLUP | Phenotypes | 0.24(0.02) | -0.01(0.10) | 0.21(0.13) | 0.29(0.06) | 0.20(0.09) |
|  |  | Adj. Phenotype | 0.21(0.03) | 0.23(0.05) | 0.18(0.11) | 0.13(0.06) | 0.19(0.08) |
|  | BayesR | Phenotypes | 0.20(0.02) | 0.10(0.05) | 0.17(0.07) | 0.20(0.07) | 0.10(0.07) |
|  |  | Adj. Phenotype | 0.20(0.03) | 0.22(0.04) | 0.19(0.07) | 0.17(0.04) | 0.23(0.07) |
|  |  |  |  |  |  |  |  |
| HCWT | BLUP | Phenotypes | 0.13(0.02) | 0.01(0.06) | 0.05(0.05) | 0.03(0.08) | 0.06(0.05) |
|  |  | Adj. Phenotype | 0.11(0.01) | 0.09(0.05) | 0.16(0.06) | 0.03(0.08) | 0.06(0.02) |
|  | GBLUP | Phenotypes | 0.35(0.03) | 0.00(0.05) | -0.05(0.08) | 0.21(0.09) | 0.22(0.07) |
|  |  | Adj. Phenotype | 0.18(0.02) | 0.17(0.04) | 0.29(0.06) | 0.18(0.08) | 0.17(0.04) |
|  | BayesR | Phenotypes | 0.21(0.03) | 0.01(0.05) | -0.01(0.06) | 0.15(0.08) | 0.14(0.06) |
|  |  | Adj. Phenotype | 0.17(0.01) | 0.17(0.03) | 0.27(0.06) | 0.12(0.09) | 0.12(0.03) |
|  |  |  |  |  |  |  |  |
| DRESS | BLUP | Phenotypes | 0.10(0.04) | -0.08(0.06) | 0.10(0.11) | 0.04(0.07) | 0.06(0.07) |
|  |  | Adj. Phenotype | 0.17(0.04) | 0.14(0.06) | 0.28(0.08) | 0.19(0.05) | 0.22(0.09) |
|  | GBLUP | Phenotypes | 0.49(0.04) | -0.01(0.04) | 0.08(0.08) | 0.38(0.04) | 0.26(0.06) |
|  |  | Adj. Phenotype | 0.20(0.04) | 0.21(0.07) | 0.20(0.10) | 0.34(0.03) | 0.26(0.04) |
|  | BayesR | Phenotypes | 0.30(0.03) | 0.04(0.03) | 0.11(0.08) | 0.35(0.05) | 0.14(0.07) |
|  |  | Adj. Phenotype | 0.23(0.03) | 0.16(0.07) | 0.22(0.07) | 0.37(0.04) | 0.22(0.05) |
|  |  |  |  |  |  |  |  |
| LMY | BLUP | Phenotypes | 0.20(0.04) | 0.20(0.10) | 0.11(0.18) | 0.21(0.10) | 0.11(0.14) |
|  |  | Adj. Phenotype | 0.18(0.04) | 0.18(0.09) | 0.14(0.19) | 0.16(0.06) | 0.22(0.05) |
|  | GBLUP | Phenotypes | 0.51(0.03) | 0.30(0.12) | 0.13(0.09) | 0.31(0.08) | 0.40(0.16) |
|  |  | Adj. Phenotype | 0.27(0.05) | 0.20(0.08) | 0.18(0.10) | 0.13(0.06) | 0.30(0.11) |
|  | BayesR | Phenotypes | 0.35(0.03) | 0.36(0.11) | 0.11(0.08) | 0.20(0.06) | 0.31(0.10) |
|  |  | Adj. Phenotype | 0.27(0.04) | 0.24(0.10) | 0.21(0.07) | 0.20(0.06) | 0.32(0.12) |
|  |  |  |  |  |  |  |  |
| IMF | BLUP | Phenotypes | 0.10(0.03) | 0.16(0.05) | 0.15(0.07) | 0.09(0.04) | 0.17(0.06) |
|  |  | Adj. Phenotype | 0.14(0.02) | 0.13(0.04) | 0.17(0.07) | 0.12(0.04) | 0.22(0.03) |
|  | GBLUP | Phenotypes | 0.38(0.03) | 0.35(0.06) | 0.37(0.07) | 0.32(0.06) | 0.26(0.05) |
|  |  | Adj. Phenotype | 0.25(0.03) | 0.23(0.07) | 0.29(0.07) | 0.33(0.05) | 0.30(0.04) |
|  | BayesR | Phenotypes | 0.27(0.03) | 0.29(0.06) | 0.34(0.07) | 0.31(0.06) | 0.26(0.04) |
|  |  | Adj. Phenotype | 0.27(0.02) | 0.24(0.06) | 0.32(0.08) | 0.35(0.04) | 0.34(0.05) |
|  |  |  |  |  |  |  |  |
| IRON | BLUP | Phenotypes | 0.18(0.03) | 0.13(0.09) | 0.05(0.10) | 0.19(0.09) | 0.20(0.10) |
|  |  | Adj. Phenotype | 0.16(0.02) | 0.09(0.06) | 0.16(0.13) | 0.20(0.04) | 0.24(0.05) |
|  | GBLUP | Phenotypes | 0.54(0.04) | 0.40(0.07) | 0.36(0.09) | 0.25(0.10) | 0.37(0.07) |
|  |  | Adj. Phenotype | 0.22(0.06) | 0.24(0.07) | 0.28(0.08) | 0.21(0.07) | 0.30(0.08) |
|  | BayesR | Phenotypes | 0.20(0.04) | 0.22(0.08) | 0.13(0.05) | 0.12(0.10) | 0.24(0.07) |
|  |  | Adj. Phenotype | 0.17(0.02) | 0.13(0.05) | 0.24(0.07) | 0.17(0.05) | 0.26(0.08) |
|  |  |  |  |  |  |  |  |
| EPA | BLUP | Phenotypes | 0.09(0.07) | 0.04(0.14) | 0.07(0.10) | -0.02(0.11) | 0.23(0.05) |
|  |  | Adj. Phenotype | 0.13(0.02) | 0.22(0.06) | -0.03(0.15) | 0.18(0.10) | 0.18(0.03) |
|  | GBLUP | Phenotypes | 0.13(0.05) | 0.07(0.15) | 0.04(0.07) | 0.12(0.14) | 0.20(0.07) |
|  |  | Adj. Phenotype | 0.14(0.03) | 0.23(0.03) | -0.04(0.14) | 0.17(0.12) | 0.20(0.06) |
|  | BayesR | Phenotypes | 0.06(0.05) | 0.03(0.12) | 0.03(0.06) | 0.06(0.13) | 0.12(0.04) |
|  |  | Adj. Phenotype | 0.11(0.05) | 0.22(0.04) | -0.03(0.14) | 0.15(0.13) | 0.18(0.05) |
|  |  |  |  |  |  |  |  |
| DPA | BLUP | Phenotypes | 0.06(0.03) | 0.13(0.08) | 0.11(0.05) | -0.12(0.08) | 0.07(0.09) |
|  |  | Adj. Phenotype | 0.09(0.05) | 0.26(0.12) | -0.08(0.09) | 0.19(0.11) | 0.10(0.09) |
|  | GBLUP | Phenotypes | 0.16(0.05) | 0.12(0.09) | 0.01(0.07) | 0.32(0.12) | 0.21(0.17) |
|  |  | Adj. Phenotype | 0.06(0.05) | 0.18(0.08) | -0.10(0.09) | 0.13(0.09) | 0.08(0.09) |
|  | BayesR | Phenotypes | 0.09(0.05) | 0.16(0.08) | -0.01(0.09) | 0.06(0.11) | 0.07(0.08) |
|  |  | Adj. Phenotype | 0.11(0.08) | 0.26(0.08) | -0.03(0.10) | 0.27(0.14) | 0.11(0.09) |

Table S2. Bias of BLUP, GBLUP and BayesR, calculated as regression of observed variables on (GEBV + polygenic), where the observed variable was either a phenotype or an adjusted phenotype.

| Trait | Method | Obs.Var. | ALL | MER | BL | PD | WS |
| --- | --- | --- | --- | --- | --- | --- | --- |
| EMD | BLUP | Phenotype | 3.60 | 1.68 | 0.96 | 0.84 | 2.08 |
|  |  | Adj. Phenotype | 1.49 | 1.98 | 0.88 | 2.53 | 0.78 |
|  | GBLUP | Phenotypes | 2.13 | 0.00 | 1.06 | 0.84 | 0.89 |
|  |  | Adj. Phenotype | 0.12 | 0.29 | 0.39 | 0.86 | 0.78 |
|  | BayesR | Phenotypes | 1.88 | 0.13 | 0.59 | 1.54 | 0.95 |
|  |  | Adj. Phenotype | 1.09 | 1.14 | 1.01 | 1.21 | 1.19 |
|  |  |  |  |  |  |  |  |
| FAT | BLUP | Phenotypes | 0.68 | 0.65 | 0.66 | 0.92 | -0.15 |
|  |  | Adj. Phenotype | 0.78 | 1.11 | 1.14 | 0.33 | 0.73 |
|  | GBLUP | Phenotypes | 1.26 | 0.16 | 1.22 | 1.58 | 1.15 |
|  |  | Adj. Phenotype | 0.76 | 0.90 | 0.75 | 0.60 | 0.72 |
|  | BayesR | Phenotypes | 1.47 | 0.79 | 1.02 | 1.22 | 0.63 |
|  |  | Adj. Phenotype | 1.04 | 1.07 | 1.19 | 0.93 | 1.19 |
|  |  |  |  |  |  |  |  |
| HCWT | BLUP | Phenotypes | 1.05 | 0.04 | 0.83 | 0.39 | 0.44 |
|  |  | Adj. Phenotype | 0.57 | 0.52 | 0.96 | 0.16 | 0.15 |
|  | GBLUP | Phenotypes | 1.15 | -0.05 | -0.36 | 1.05 | 0.97 |
|  |  | Adj. Phenotype | 0.37 | 0.61 | 1.21 | 0.56 | 0.54 |
|  | BayesR | Phenotypes | 1.35 | 0.05 | -0.17 | 1.08 | 0.93 |
|  |  | Adj. Phenotype | 0.72 | 0.81 | 1.45 | 0.53 | 0.60 |
|  |  |  |  |  |  |  |  |
| DRESS | BLUP | Phenotypes | 1.06 | -1.08 | 0.76 | 0.15 | 0.52 |
|  |  | Adj. Phenotype | 1.03 | 0.94 | 2.39 | 1.07 | 1.24 |
|  | GBLUP | Phenotypes | 1.30 | -0.06 | 0.25 | 1.43 | 1.00 |
|  |  | Adj. Phenotype | 0.33 | 0.57 | 0.59 | 0.77 | 0.58 |
|  | BayesR | Phenotypes | 1.75 | 0.21 | 0.63 | 1.99 | 0.85 |
|  |  | Adj. Phenotype | 0.83 | 0.60 | 0.96 | 1.32 | 0.84 |
|  |  |  |  |  |  |  |  |
| LMY | BLUP | Phenotypes | 1.68 | 2.32 | 1.75 | 2.31 | 0.37 |
|  |  | Adj. Phenotype | 1.08 | 1.59 | 1.67 | 1.22 | 2.05 |
|  | GBLUP | Phenotypes | 2.02 | 1.41 | 0.50 | 1.49 | 1.62 |
|  |  | Adj. Phenotype | 0.77 | 0.66 | 0.61 | 0.50 | 1.11 |
|  | BayesR | Phenotypes | 2.09 | 2.27 | 0.53 | 1.42 | 1.95 |
|  |  | Adj. Phenotype | 1.18 | 1.15 | 0.87 | 1.05 | 2.32 |
|  |  |  |  |  |  |  |  |
| IMF | BLUP | Phenotypes | 0.81 | 1.35 | 1.18 | 0.76 | 1.02 |
|  |  | Adj. Phenotype | 0.84 | 0.72 | 0.99 | 0.81 | 1.26 |
|  | GBLUP | Phenotypes | 1.19 | 1.37 | 1.24 | 1.01 | 0.86 |
|  |  | Adj. Phenotype | 0.62 | 0.72 | 0.75 | 0.86 | 0.82 |
|  | BayesR | Phenotypes | 1.11 | 1.40 | 1.37 | 1.11 | 1.02 |
|  |  | Adj. Phenotype | 0.85 | 0.90 | 1.02 | 1.03 | 1.09 |
|  |  |  |  |  |  |  |  |
| IRON | BLUP | Phenotypes | 1.75 | 1.51 | 0.56 | 2.05 | 1.54 |
|  |  | Adj. Phenotype | 1.13 | 0.85 | 1.86 | 1.45 | 1.65 |
|  | GBLUP | Phenotypes | 2.13 | 2.10 | 2.19 | 1.22 | 1.82 |
|  |  | Adj. Phenotype | 0.75 | 1.13 | 1.26 | 0.84 | 1.41 |
|  | BayesR | Phenotypes | 1.59 | 1.78 | 1.27 | 0.98 | 1.69 |
|  |  | Adj. Phenotype | 1.02 | 0.97 | 1.74 | 0.94 | 1.66 |
|  |  |  |  |  |  |  |  |
| EPA | BLUP | Phenotypes | 1.61 | 0.55 | 1.43 | -1.63 | 4.85 |
|  |  | Adj. Phenotype | 1.28 | 1.85 | -0.20 | 1.67 | -0.42 |
|  | GBLUP | Phenotypes | 2.11 | 1.24 | 0.94 | 0.98 | 4.07 |
|  |  | Adj. Phenotype | 1.08 | 1.51 | -0.16 | 1.27 | 1.64 |
|  | BayesR | Phenotypes | 1.10 | 0.75 | 0.61 | 0.41 | 2.43 |
|  |  | Adj. Phenotype | 0.93 | 1.48 | 0.08 | 1.20 | -0.22 |
|  |  |  |  |  |  |  |  |
| DPA | BLUP | Phenotypes | 1.22 | 2.38 | 2.50 | -3.80 | 1.76 |
|  |  | Adj. Phenotype | 0.85 | 2.12 | -1.72 | 2.44 | 0.99 |
|  | GBLUP | Phenotypes | 1.55 | 1.63 | -0.26 | 3.17 | 0.80 |
|  |  | Adj. Phenotype | 0.36 | 1.06 | -0.79 | 1.16 | 0.29 |
|  | BayesR | Phenotypes | 1.67 | 2.06 | 0.00 | 0.73 | 1.91 |
|  |  | Adj. Phenotype | 1.03 | 2.04 | -0.41 | 2.90 | 1.15 |

Table S3. Mean of top 10 genomic relationships of validation animals to reference

| Trait | ALL | MER | BL | PD | WS |
| --- | --- | --- | --- | --- | --- |
| EMD | 0.114223 | 0.103339 | 0.126898 | 0.121745 | 0.11796 |
| FAT | 0.114223 | 0.103339 | 0.126898 | 0.121745 | 0.11796 |
| HCWT | 0.114223 | 0.103339 | 0.126898 | 0.121745 | 0.11796 |
| DRESS | 0.119941 | 0.102397 | 0.119262 | 0.125761 | 0.124638 |
| LMY | 0.131317 | 0.12307 | 0.139489 | 0.134769 | 0.133622 |
| IMF | 0.162366 | 0.127634 | 0.163235 | 0.157663 | 0.145604 |
| IRON | 0.146656 | 0.167752 | 0.157738 | 0.144536 | 0.14541 |
| EPA | 0.142456 | 0.150674 | 0.153811 | 0.134067 | 0.144245 |
| DPA | 0.140545 | 0.151163 | 0.15115 | 0.139383 | 0.131903 |

Table S4. Number of validation animals (SE) per sire breed across subsets used for calculating accuracy, bias and relationships to reference.

| Trait | ALL | MER | BL | PD | WS |
| --- | --- | --- | --- | --- | --- |
| EMD | 512(36) | 78(6) | 59(11) | 149(23) | 117(20) |
| FAT | 497(35) | 77(6) | 57(10) | 145(23) | 113(20) |
| HCWT | 517(37) | 79(7) | 59(11) | 151(24) | 118(21) |
| DRESS | 497(21) | 79(8) | 56(12) | 148(15) | 114(16) |
| LMY | 525(35) | 80(8) | 58(12) | 160(26) | 121(25) |
| IMF | 516(32) | 80(10) | 130(60) | 153(20) | 119(25) |
| IRON | 513(33) | 80(8) | 63(14) | 151(27) | 119(25) |
| EPA | 511(41) | 86(12) | 83(13) | 167(25) | 116(30) |
| DPA | 511(34) | 86(16) | 83(13) | 167(32) | 116(23) |

Table S5. Description of potential candidate genes within 0.5Mb of SNP > 0.05SD effects.

| **Gene Name** | **Full Name** | **Pos** | **Species** |
| --- | --- | --- | --- |
| ROBO2 | Roundabout homolog 2 | OAR1:143271321..143464499 | Human |
| SATB1 | DNA-binding protein SATB1 | [OAR1:274131837..274204893](http://www.livestockgenomics.csiro.au/cgi-bin/gbrowse/oarv2.0?name=OAR1:274131837..274204893) | Bos Taurus |
| TSC21 | Protein TSC21 | [OAR3:58538432..58542007](http://www.livestockgenomics.csiro.au/cgi-bin/gbrowse/oarv2.0?name=OAR3:58538432..58542007) | Bos Taurus, |
| EIF2AK3 | Eukaryotic translation initiation factor 2-alpha kinase 3 | [OAR3:58568969..58641017](http://www.livestockgenomics.csiro.au/cgi-bin/gbrowse/oarv2.0?name=OAR3:58568969..58641017) | Bos Taurus |
| RPIA | Ribose-5-phosphate isomerase | [OAR3:58720873..58743861](http://www.livestockgenomics.csiro.au/cgi-bin/gbrowse/oarv2.0?name=OAR3:58720873..58743861) | Bos Taurus |
| IGK | IGK protein | [OAR3:58786078..58862200](http://www.livestockgenomics.csiro.au/cgi-bin/gbrowse/oarv2.0?name=OAR3:58786078..58862200) | Bos Taurus |
| PSD4 | PH and SEC7 domain-containing protein 4 | [OAR3:59126249..59143286](http://www.livestockgenomics.csiro.au/cgi-bin/gbrowse/oarv2.0?name=OAR3:59126249..59143286) | Human |
| IL1RN | Interleukin-1 receptor antagonist protein | [OAR3:59191431..59197070](http://www.livestockgenomics.csiro.au/cgi-bin/gbrowse/oarv2.0?name=OAR3:59191431..59197070) | Bos Taurus |
| IL1F10 | Interleukin-1 family member 10 | [OAR3:59267183..59270574](http://www.livestockgenomics.csiro.au/cgi-bin/gbrowse/oarv2.0?name=OAR3:59267183..59270574) | Human |
| IL1F5 | Interleukin 1 family, member 5 (Delta) | [OAR3:59285427..59288377](http://www.livestockgenomics.csiro.au/cgi-bin/gbrowse/oarv2.0?name=OAR3:59285427..59288377) | Bos Taurus |
| SLC20A1 | Solute carrier family 20 (Phosphate transporter), member 1 | OAR3:59692220..59706537 | Bos Taurus |
| CHCHD5 | Coiled-coil-helix-coiled-coil-helix domain containing 5 | [OAR3:59778816..59779209](http://www.livestockgenomics.csiro.au/cgi-bin/gbrowse/oarv2.0?name=OAR3:59778816..59779209) | Bos Taurus |
| POLR1B | DNA-directed RNA polymerase | [OAR3:59823194..59853618](http://www.livestockgenomics.csiro.au/cgi-bin/gbrowse/oarv2.0?name=OAR3:59823194..59853618) | Bos Taurus |
| TTL | Tubulin--tyrosine ligase | [OAR3:59823194..59853618](http://www.livestockgenomics.csiro.au/cgi-bin/gbrowse/oarv2.0?name=OAR3:59823194..59853618) | Bos Taurus |
| NCK2 | NCK2 protein | [OAR3:60195601..60221497](http://www.livestockgenomics.csiro.au/cgi-bin/gbrowse/oarv2.0?name=OAR3:60195601..60221497) | Bos Taurus |
| Augurin |  | [OAR3:60300197..60309219](http://www.livestockgenomics.csiro.au/cgi-bin/gbrowse/oarv2.0?name=OAR3:60300197..60309219) | Bos Taurus |
| cDNA FLJ78230 |  | [OAR3:60352301..60391713](http://www.livestockgenomics.csiro.au/cgi-bin/gbrowse/oarv2.0?name=OAR3:60352301..60391713) | Human |
| ST6GalII | Beta-galactoside alpha-2,6-sialyltransferase | [OAR3:60537181..60561570](http://www.livestockgenomics.csiro.au/cgi-bin/gbrowse/oarv2.0?name=OAR3:60537181..60561570) | Bos Taurus |
| PPM1K | Protein phosphatase 1K, mitochondrial | [OAR6:36455393..36469064](http://www.livestockgenomics.csiro.au/cgi-bin/gbrowse/oarv2.0?name=OAR6:36455393..36469064) | Bos Taurus |
| ABCG2 | ATP-binding cassette sub-family G member 2 | [OAR6:36565022..36611117](http://www.livestockgenomics.csiro.au/cgi-bin/gbrowse/oarv2.0?name=OAR6:36565022..36611117) | Ovis Aries |
| PKD2 | Polycystin-2 | [OAR6:36620660..36674500](http://www.livestockgenomics.csiro.au/cgi-bin/gbrowse/oarv2.0?name=OAR6:36620660..36674500) | Bos Taurus |
| SPP1 | Osteopontin | [OAR6:36709309..36714661](http://www.livestockgenomics.csiro.au/cgi-bin/gbrowse/oarv2.0?name=OAR6:36709309..36714661) | Ovis Aries |
| MEPE | Extracellular phosphoglycoprotein with ASARM motif (Bone) | [OAR6:36862324..36871855](http://www.livestockgenomics.csiro.au/cgi-bin/gbrowse/oarv2.0?name=OAR6:36862324..36871855) | Human |
| IBSP | Bone sialoprotein 2 | [OAR6:36896627..36907248](http://www.livestockgenomics.csiro.au/cgi-bin/gbrowse/oarv2.0?name=OAR6:36896627..36907248) | Bos Taurus |
| LAP3 | Cytosol aminopeptidase | [OAR6:37159078..37180053](http://www.livestockgenomics.csiro.au/cgi-bin/gbrowse/oarv2.0?name=OAR6:37159078..37180053) | Bos Taurus |
| FAM184A | Family with sequence similarity 184, member A | [OAR6:37202919..37321059](http://www.livestockgenomics.csiro.au/cgi-bin/gbrowse/oarv2.0?name=OAR6:37202919..37321059) | Human |
| DCAF16 | DDB1- and CUL4-associated factor 16 | [OAR6:37342592..37343242](http://www.livestockgenomics.csiro.au/cgi-bin/gbrowse/oarv2.0?name=OAR6:37342592..37343242) | Human |
| NCAPG | Condensin complex subunit 3 | [OAR6:37353060..37397804](http://www.livestockgenomics.csiro.au/cgi-bin/gbrowse/oarv2.0?name=OAR6:37353060..37397804) | Bos Taurus |
| LCORL | Ligand-dependent nuclear receptor corepressor-like protein | [OAR6:37429189..37516231](http://www.livestockgenomics.csiro.au/cgi-bin/gbrowse/oarv2.0?name=OAR6:37429189..37516231) | Human |
| CRYBA1 | Beta-crystallin A3 | [OAR11:20247706..20254476](http://www.livestockgenomics.csiro.au/cgi-bin/gbrowse/oarv2.0?name=OAR11:20247706..20254476) | Bos Taurus |
| NUFIP2 | Nuclear fragile X mental retardation-interacting protein 2 | [OAR11:20263704..20282766](http://www.livestockgenomics.csiro.au/cgi-bin/gbrowse/oarv2.0?name=OAR11:20263704..20282766) | Human |
| TAOK1 | Serine/threonine-protein kinase TAO1 | [OAR11:20405602..20458608](http://www.livestockgenomics.csiro.au/cgi-bin/gbrowse/oarv2.0?name=OAR11:20405602..20458608) | Human |
| GIT1 | ARF GTPase-activating protein GIT1 | [OAR11:20490262..20499430](http://www.livestockgenomics.csiro.au/cgi-bin/gbrowse/oarv2.0?name=OAR11:20490262..20499430) | Human |
| ANKRD13B | Ankyrin repeat domain-containing protein 13B | [OAR11:20519151..20524549](http://www.livestockgenomics.csiro.au/cgi-bin/gbrowse/oarv2.0?name=OAR11:20519151..20524549) | Human |
| SSH2 | Protein phosphatase Slingshot homolog 2 | [OAR11:20543757..20645282](http://www.livestockgenomics.csiro.au/cgi-bin/gbrowse/oarv2.0?name=OAR11:20543757..20645282) | Human |
| EFCAB5 | EF-hand calcium-binding domain-containing protein 5 | [OAR11:20837315..20909102](http://www.livestockgenomics.csiro.au/cgi-bin/gbrowse/oarv2.0?name=OAR11:20837315..20909102) | Human |
| NSRP1 | Nuclear speckle splicing regulatory protein 1 | [OAR11:20915750..20957531](http://www.livestockgenomics.csiro.au/cgi-bin/gbrowse/oarv2.0?name=OAR11:20915750..20957531) | Bos Taurus |
| SLC6A4 | Sodium-dependent serotonin transporter | [OAR11:20965846..20985828](http://www.livestockgenomics.csiro.au/cgi-bin/gbrowse/oarv2.0?name=OAR11:20965846..20985828) | Ovis Aries |
| BLMH | Bleomycin hydrolase | [OAR11:21010794..21051986](http://www.livestockgenomics.csiro.au/cgi-bin/gbrowse/oarv2.0?name=OAR11:21010794..21051986) | Human |
| CPD | Carboxypeptidase D | [OAR11:21142529..21211550](http://www.livestockgenomics.csiro.au/cgi-bin/gbrowse/oarv2.0?name=OAR11:21142529..21211550) | Human |
| Chymotrypsinogen A | Chymotrypsinogen A | [OAR14:1913484..1916935](http://www.livestockgenomics.csiro.au/cgi-bin/gbrowse/oarv2.0?name=OAR14:1913484..1916935) | Bos Taurus |
| BCAR1 | Breast cancer anti-estrogen resistance protein 1 | [OAR14:1923576..1936716](http://www.livestockgenomics.csiro.au/cgi-bin/gbrowse/oarv2.0?name=OAR14:1923576..1936716) | Bos Taurus |
| CFDP1 | Craniofacial development protein 1 | [OAR14:1974602..2074282](http://www.livestockgenomics.csiro.au/cgi-bin/gbrowse/oarv2.0?name=OAR14:1974602..2074282) | Bos Taurus |
| CFDP2 | Craniofacial development protein 2 | [OAR14:2116318..2134419](http://www.livestockgenomics.csiro.au/cgi-bin/gbrowse/oarv2.0?name=OAR14:2116318..2134419) | Bos Taurus |
| TMEM170A | Transmembrane protein 170A | [OAR14:2154810..2157564](http://www.livestockgenomics.csiro.au/cgi-bin/gbrowse/oarv2.0?name=OAR14:2154810..2157564) | Human |
| [BVDV](http://www.uniprot.org/taxonomy/11099) | Bovine viral diarrhea virus (BVDV) (Mucosal disease virus) | [OAR14:2220109..2230514](http://www.livestockgenomics.csiro.au/cgi-bin/gbrowse/oarv2.0?name=OAR14:2220109..2230514) | Bos Taurus |
| ADAT1 | Adenosine deaminase, tRNA-specific 1 | [OAR14:2257464..2268982](http://www.livestockgenomics.csiro.au/cgi-bin/gbrowse/oarv2.0?name=OAR14:2257464..2268982) | Bos Taurus |
| KARS | Lysyl-tRNA synthetase | [OAR14:2274411..2286653](http://www.livestockgenomics.csiro.au/cgi-bin/gbrowse/oarv2.0?name=OAR14:2274411..2286653) | Bos Taurus |
| CNTNAP4 | Contactin-associated protein-like 4 | [OAR14:2799238..3042826](http://www.livestockgenomics.csiro.au/cgi-bin/gbrowse/oarv2.0?name=OAR14:2799238..3042826) | Human |
| HEATR3 | HEAT repeat containing 3 | [OAR14:17662881..17696251](http://www.livestockgenomics.csiro.au/cgi-bin/gbrowse/oarv2.0?name=OAR14:17662881..17696251) | Bos Taurus |
| PAPD5 | [Uncharacterized protein](http://www.ensembl.org/id/ENSP00000396995) | [OAR14:17793357..17807280](http://www.livestockgenomics.csiro.au/cgi-bin/gbrowse/oarv2.0?name=OAR14:17793357..17807280) | Human |
| ADCY7 | Adenylate cyclase type 7 | [OAR14:17860908..17888080](http://www.livestockgenomics.csiro.au/cgi-bin/gbrowse/oarv2.0?name=OAR14:17860908..17888080) | Bos Taurus |
| BRD7 | Bromodomain-containing protein 7 | [OAR14:17891893..17940079](http://www.livestockgenomics.csiro.au/cgi-bin/gbrowse/oarv2.0?name=OAR14:17891893..17940079) | Bos Taurus |
| NKD1 | Protein naked cuticle homolog 1 | [OAR14:18115168..18206771](http://www.livestockgenomics.csiro.au/cgi-bin/gbrowse/oarv2.0?name=OAR14:18115168..18206771) | Bos Taurus |
| NX20 | Sorting nexin-20 | [OAR14:18241898..18245611](http://www.livestockgenomics.csiro.au/cgi-bin/gbrowse/oarv2.0?name=OAR14:18241898..18245611) | Bos Taurus |
| NOD2 | Nucleotide-binding oligomerization domain-containing protein 2 | [OAR14:18273991..18300766](http://www.livestockgenomics.csiro.au/cgi-bin/gbrowse/oarv2.0?name=OAR14:18273991..18300766) | Bos Taurus |
| CYLD | Ubiquitin carboxyl-terminal hydrolase CYLD | [OAR14:18322667..18377630](http://www.livestockgenomics.csiro.au/cgi-bin/gbrowse/oarv2.0?name=OAR14:18322667..18377630) | Bos Taurus |

Figure S1. Plots of absolute marker effects in adjusted phenotypic SD for all traits
